# Expanding the Evidence on the Safety and Efficiency of 2-Way Text Messaging–Based Telehealth for Voluntary Medical Male Circumcision Follow-up Compared With In-Person Reviews: Randomized Controlled Trial in Rural and Urban South Africa

**DOI:** 10.2196/42111

**Published:** 2023-05-09

**Authors:** Caryl Feldacker, Jacqueline Pienaar, Beatrice Wasunna, Felex Ndebele, Calsile Khumalo, Sarah Day, Hannock Tweya, Femi Oni, Maria Sardini, Binod Adhikary, Evelyn Waweru, Mourice Barasa Wafula, Anna Dixon, Krishna Jafa, Yanfang Su, Kenneth Sherr, Geoffrey Setswe

**Affiliations:** 1 Department of Global Health University of Washington Seattle, WA United States; 2 International Training and Education Center for Health Department of Global Health University of Washington Seattle, WA United States; 3 Aurum Institute Johannesburg South Africa; 4 Medic Nairobi Kenya; 5 Centre for HIV-AIDS Prevention Studies (CHAPS) Johannesburg South Africa; 6 Faculty of Health Sciences University of Cape Town Cape Town South Africa; 7 Medic Kathmandu Nepal; 8 Medic San Francisco, CA United States; 9 Department of Health Studies University of South Africa (UNISA) Pretoria South Africa

**Keywords:** SMS text messaging–based telehealth, 2-way texting, voluntary medical male circumcision, South Africa, mobile health, mHealth for quality improvement, digital health innovation in low- and middle-income countries, male engagement in care, COVID-19, mobile phone

## Abstract

**Background:**

There is a dearth of high-quality evidence from digital health interventions in routine program settings in low- and middle-income countries. We previously conducted a randomized controlled trial (RCT) in Zimbabwe, demonstrating that 2-way texting (2wT) was safe and effective for follow-up after adult voluntary medical male circumcision (VMMC).

**Objective:**

To demonstrate the replicability of 2wT, we conducted a larger RCT in both urban and rural VMMC settings in South Africa to determine whether 2wT improves adverse event (AE) ascertainment and, therefore, the quality of follow-up after VMMC while reducing health care workers’ workload.

**Methods:**

A prospective, unblinded, noninferiority RCT was conducted among adult participants who underwent VMMC with cell phones randomized in a 1:1 ratio between 2wT and control (routine care) in North West and Gauteng provinces. The 2wT participants responded to a daily SMS text message with in-person follow-up only if desired or an AE was suspected. The control group was requested to make in-person visits on postoperative days 2 and 7 as per national VMMC guidelines. All participants were asked to return on postoperative day 14 for study-specific review. Safety (cumulative AEs ≤day 14 visit) and workload (number of in-person follow-up visits) were compared. Differences in cumulative AEs were calculated between groups. Noninferiority was prespecified with a margin of −0.25%. The Manning score method was used to calculate 95% CIs.

**Results:**

The study was conducted between June 7, 2021, and February 21, 2022. In total, 1084 men were enrolled (2wT: n=547, 50.5%, control: n=537, 49.5%), with near-equal proportions of rural and urban participants. Cumulative AEs were identified in 2.3% (95% CI 1.3-4.1) of 2wT participants and 1.0% (95% CI 0.4-2.3) of control participants, demonstrating noninferiority (1-sided 95% CI −0.09 to ∞). Among the 2wT participants, 11 AEs (9 moderate and 2 severe) were identified, compared with 5 AEs (all moderate) among the control participants—a nonsignificant difference in AE rates (*P*=.13). The 2wT participants attended 0.22 visits, and the control participants attended 1.34 visits—a significant reduction in follow-up visit workload (*P*<.001). The 2wT approach reduced unnecessary postoperative visits by 84.8%. Daily response rates ranged from 86% on day 3 to 74% on day 13. Among the 2wT participants, 94% (514/547) responded to ≥1 daily SMS text messages over 13 days.

**Conclusions:**

Across rural and urban contexts in South Africa, 2wT was noninferior to routine in-person visits for AE ascertainment, demonstrating 2wT safety. The 2wT approach also significantly reduced the follow-up visit workload, improving efficiency. These results strongly suggest that 2wT provides quality VMMC follow-up and should be adopted at scale. Adaptation of the 2wT telehealth approach to other acute follow-up care contexts could extend these gains beyond VMMC.

**Trial Registration:**

ClinicalTrials.gov NCT04327271; https://www.clinicaltrials.gov/ct2/show/NCT04327271

## Introduction

### Background

In sub-Saharan Africa (SSA), direct provider-to-client communication approaches, including 2-way texting (2wT) and telehealth, are being deployed in HIV prevention, treatment, and care services with promising results, to improve patient engagement in care, including adherence [1-6]. However, not all interventions are effective in all populations at all times [7]. Rapid responses to COVID-19 accelerated telehealth innovation in SSA to maintain critical service delivery while reducing the spread of the infection [8-12]. However, despite consistent growth in the digital health arena and available tools to guide the assessment of mobile health (mHealth) interventions [13-15], rigorous evidence of mHealth success cannot keep pace with the speed of, and demand for, innovation. A gap remains in high-quality evidence on successful mHealth approaches in routine program settings in SSA [16-18]. A greater understanding of where, when, how, and for whom a digital intervention works is needed to address critical equity issues in mHealth access [19,20] and inform mHealth impact at scale [21-24]. This is particularly important for males who may find using digital health more appealing than engaging in traditional care [25, 26]. With the global progress toward ending AIDS by 2030 threatened [27], additional effort is needed to improve the identification and replication of effective digital health interventions that increase access to critical HIV prevention, treatment, and care services.

Since 2007, voluntary medical male circumcision (VMMC) has been recognized by the Joint United Nations Programme on HIV/AIDS and World Health Organization (WHO) as a critical component of combination HIV prevention, reaching almost 30 million males in SSA by 2022 [28]. To cement the progress in HIV prevention, robust political and financial support from Ministries of Health and donor agencies, including the US President’s Emergency Plan for AIDS Relief (PEPFAR), continues to push for VMMC, with the goal of reaching 80% of eligible males in SSA [27,29-34]. VMMC programs follow global guidelines on service delivery to ensure the delivery of quality VMMC care [35]. Routine VMMC monitoring and evaluation indicators encourage the early diagnosis of adverse events (AEs) and ensure quality data for decision-making. In addition to a number of VMMC procedures, core monitoring and evaluation indicators measure the quality of care, including the number of clients with ≥1 postoperative follow-up visits within 14 days and the number of AEs by severity (moderate or severe) [3,33,36]. Over a decade of data show that VMMC is safe: across large-volume VMMC programs in SSA, the mean combined moderate and severe AE rate is below 2% [37-47], the commonly accepted threshold for safety [48]. Adherence to recommended in-person postoperative reviews by a clinician remains a PEPFAR priority [3,49]. However, with low AE rates, making postoperative reviews a requirement for all males may deter VMMC uptake and waste time for both providers and patients [50]. Moreover, the reported follow-up rates may be overestimated, and men with potential AEs may still be missed [51].

Requiring postoperative visits for all males when the risk of AEs is low suggests program inefficiencies at scale, making VMMC an excellent candidate for digital health innovation. Therefore, in 2019, the International Training and Education Center for Health at the University of Washington conducted a randomized controlled trial (RCT) in Harare, Zimbabwe, to test 2-way texting (2wT)–based telehealth for postoperative follow-up after VMMC [52]. The 2wT approach allowed males healing without complication to opt out of routine postoperative visits while engaging males with concerns in 2-way SMS text messaging with a VMMC nurse who referred them to in-person visits when needed or desired. The 2wT approach maintained patient safety by focusing on those with potential AEs while reducing unnecessary postoperative visits by 85% [50]. The 2wT approach saved US $2.0 per patient [53] and was highly acceptable to both patients and providers [54]. Results from the adaptation and scale-up of the 2wT approach in Zimbabwe from RCT to routine VMMC service delivery show similar safety and efficiency advantages, supporting its continued expansion for males aged ≥15 years [55]. An important implementation science research question is as follows: Could 2wT be replicated and scaled in different routine program settings?

In South Africa, with support from the PEPFAR, approximately 500,000 VMMCs are conducted annually as part of the National Department of Health (NDoH) HIV prevention efforts [31,56]. At this level of productivity and with low AE rates [44], >400,000 unnecessary VMMC reviews may be conducted annually. Urban sites are overwhelmed by high VMMC and review volumes, especially during seasonal high-demand periods. In rural sites, where 90% of VMMCs occur, follow-up requires multiple, sometimes multi-hour trips, eroding health care worker (HCW) productivity. The reported AE rates in South Africa are low, but AE underreporting is also common, reducing the quality of VMMC services and compromising patient care [57]. South Africa’s ambitious VMMC goals and health system constraints, combined with good cell phone coverage and existing mHealth efforts [58-60], converge to suggest that 2wT’s impact would be substantial. Greater efficiency could compensate for VMMC losses owing to the COVID-19 pandemic, reduce unnecessary costs, and maintain the quality of services.

### Rationale of This Study

To amplify and strengthen previous 2wT findings via adaptation and replication in a new country and new context, the International Training and Education Center for Health, Aurum Institute, Centre for HIV-AIDS Prevention Studies (CHAPS), and technology partner Medic conducted a 2wT RCT in routine VMMC settings in both urban and rural areas in South Africa. In this primary analysis, our objective was to determine whether 2wT improved the ascertainment of AEs and, therefore, the quality of post-VMMC follow-up while reducing HCWs’ workload. We conducted an unblinded, noninferiority RCT comparing two markers of VMMC quality: (1) moderate or severe AE rate ≤day 14 after VMMC and (2) the mean number of in-person postoperative follow-ups in the control and intervention arms. We hypothesized that 2wT would reduce the follow-up visit workload and improve AE ascertainment, reaffirming the evidence that 2wT-based VMMC follow-up is both safe and efficient. If confirmed, this additional evidence for 2wT’s advantages would support efforts to scale 2wT for VMMC follow-up in South Africa and beyond while also supporting the possibility of using 2wT-based telehealth for other acute or postoperative follow-up in routine low- and middle-income country settings.

## Methods

### Technology Overview

#### Digital Tool Design and Development

The 2wT approach is SMS text messaging–based conversational messaging between HCWs and clients. For this study, we adapted the Zimbabwe 2wT digital app that was built on the Community Health Toolkit (CHT) [61], an open-source global public good [62] that is highly secure, highly configurable, runs offline, supports use in multiple languages, and has >40,000 users across 16 countries [63-66]. Medic, a nonprofit organization, is the technical steward of the CHT. The 2wT app can be accessed on desktop computers, tablets, and mobile devices with or without an internet connection [50,55]. The CHT offers advantages in portability via local hosting and the ability to engage across multiple community health system hierarchies (districts, facilities, supervisors, HCWs, and patients). The CHT allows integration with health information systems (District Health Information Software 2) and facility-based electronic medical record systems such as OpenMRS. Engagement with the wider digital health literature and community is core to 2wT’s design, implementation, scale-up, and replication in other settings. The 2wT app aligns with the client-, provider-, manager-, and data-focused components of the WHO Classification of Digital Health Interventions [67].

**Figure 1:**
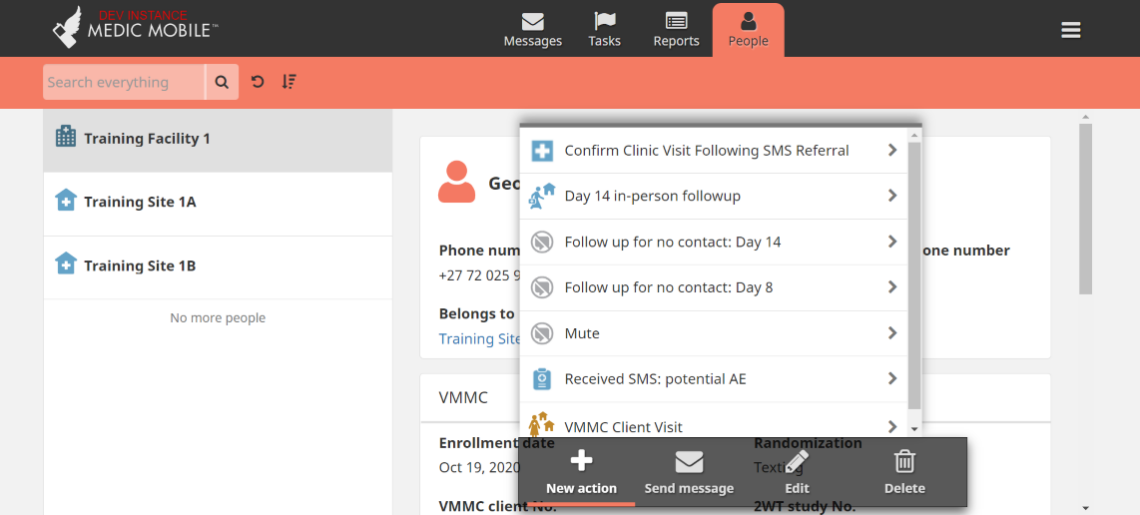
Community Health Toolkit People tab (CC BY 4.0 [55]). AE: adverse event; VMMC: voluntary medical male circumcision.

**Figure 2:**
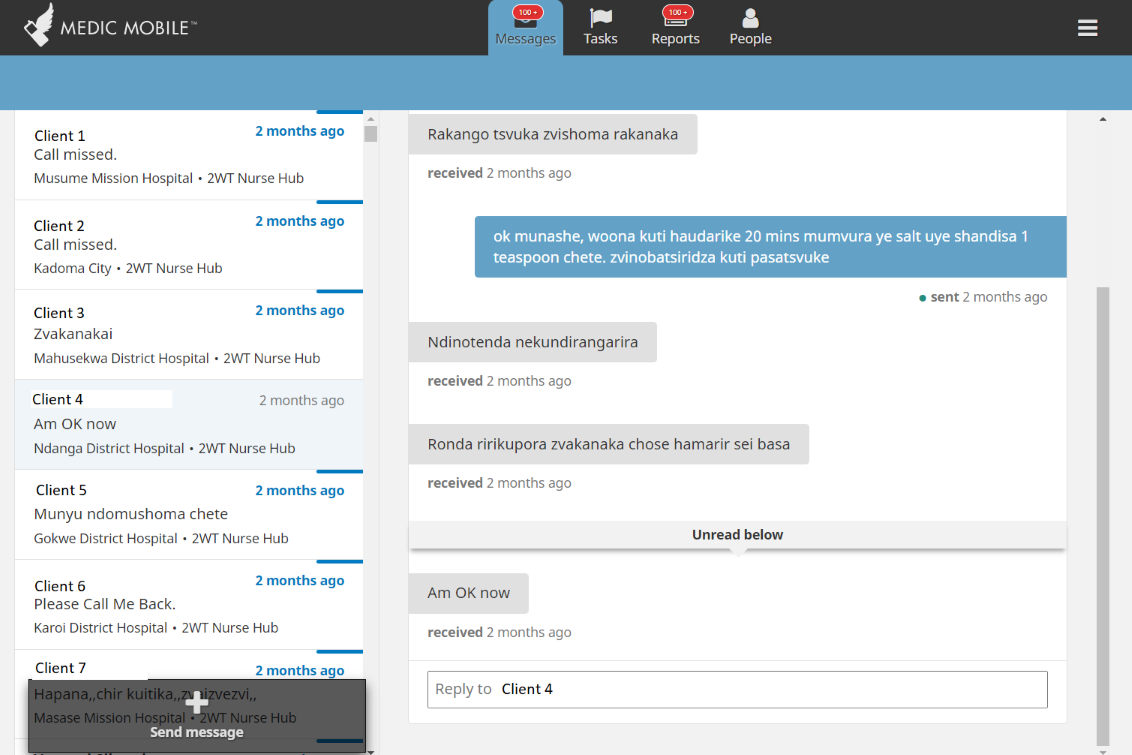
Community Health Toolkit Messages tab (CC BY 4.0 [55]).

**Figure 3:**
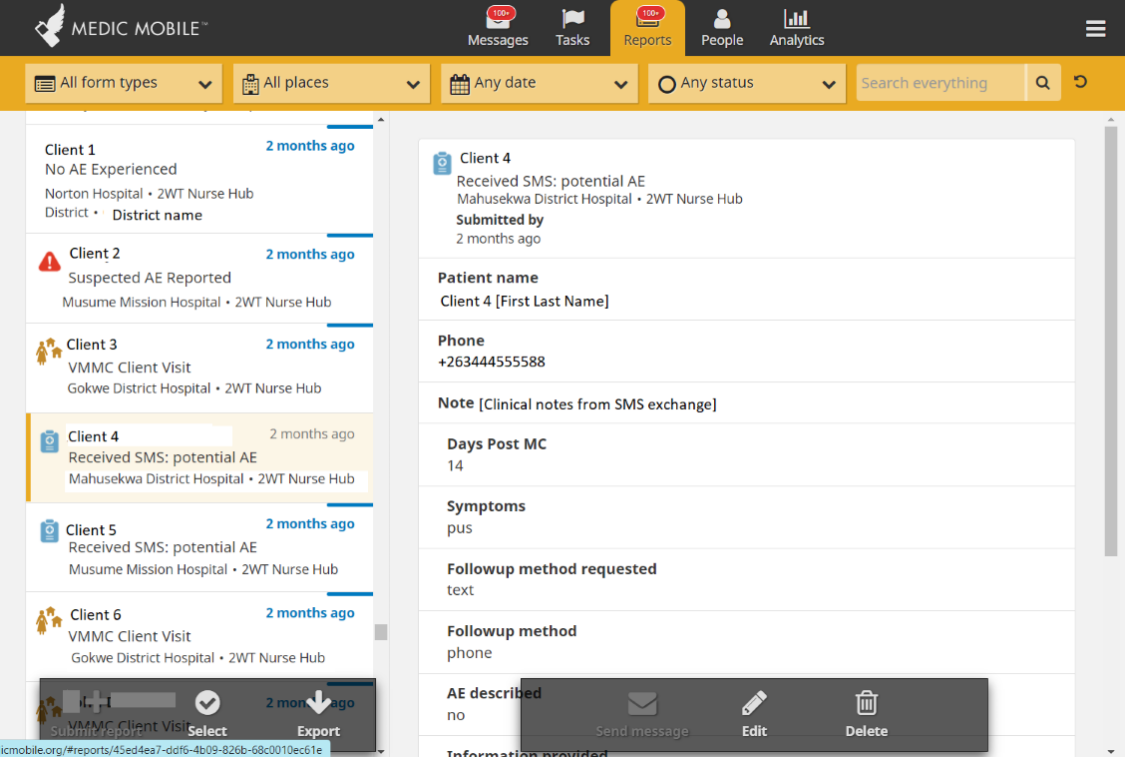
Community Health Toolkit Reports tab (CC BY 4.0 [55]). 2wT: 2-way texting; AE: adverse event; MC: male circumcision; VMMC: voluntary medical male circumcision.

We conducted Zoom (Zoom Video Communications)-based and in-person user acceptance testing and feedback sessions with patients and providers from routine VMMC program settings. The People, Messages, and Reports tabs (Figures 1-3) were updated through an iterative human-centered design process [68,69] that solicited user feedback from HCWs, nurses, and managers from all study sites to reflect local preferences and priorities, in accordance with the Principles of Digital Development [70]. SMS text message timing and content in English, isiZulu, and Setswana were pretested, modified, and optimized with clients, whereas similar feedback cycles with HCWs informed improvements for naming tasks and 2wT-based documentation. In March 2021, feedback from a small pilot with 50 males enrolled in texting further improved language clarity, smoothed patient referrals, and simplified 2wT reporting to reduce data duplication between the study and routine reporting forms. The pilot feedback also showed that clients often had 2 primary phone numbers. Improvements made before the launch of the study allowed clients to register for 2wT with multiple phone numbers from dual SIM cards or across multiple mobile networks, improving the data quality and continuity of client follow-up.

#### 2wT System Architecture

**Figure 4:**
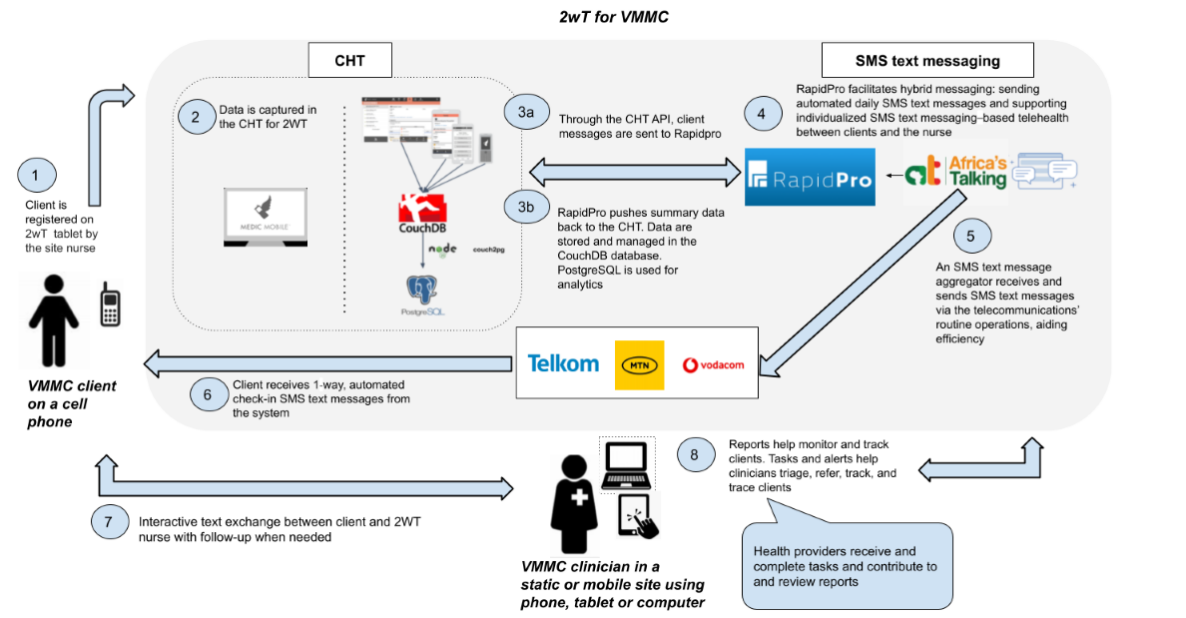
System architecture of 2-way texting (2wT) for voluntary medical male circumcision (VMMC) postoperative care. API: application programming interface; CHT: Community Health Toolkit.

For this study, we enrolled 2wT clients directly in the VMMC app (running on the CHT) and stored and managed data using CouchDB and a PostgreSQL database system (Figure 4). We sent the clients SMS text messages via RapidPro [71], an open-source software developed by the United Nations International Children's Emergency Fund (UNICEF), to create more streamlined 2-way messaging flows and benefit from default integrations with a wide range of messaging platforms. An SMS text message aggregator service and a short code (Africa’s Talking) channel linked to RapidPro streamlined the incoming and outgoing 2wT SMS text messages.

### Study Design

We conducted a 2-arm, prospective, unblinded, noninferiority RCT in 3 urban VMMC facilities in the Ekurhuleni district (Gauteng province) and 4 rural VMMC outreach sites in the Bojanala district (North West province) in South Africa. We purposively selected high–VMMC volume sites in urban and rural locations within 1 day of travel from the Aurum headquarters to ease supervision, represent different service delivery models, and consider limited study resources. The unit of randomization was individuals receiving VMMC. The participants were randomized in a 1:1 ratio and divided equally into urban and rural participants. Across all settings, we compared 2 groups of adult participants with cell phones: (1) participants who received routine care via in-person follow-up visits on days 2 and 7 (control) and (2) participants who received and responded to a daily SMS text message (2wT). The 2wT group had in-person follow-up only if desired or if an AE was suspected.

### Eligibility

Men were eligible for enrollment if they (1) were aged ≥18 years, (2) possessed a cell phone at the time of enrollment, (3) provided contact information (phone, address, and next of kin), (4) had surgical VMMC, (5) had no intraoperative AE on the day of enrollment, (6) were willing to follow NDoH VMMC protocols, (7) were willing to come to the clinic on day 14 after VMMC, (8) were willing to respond to a questionnaire administered via SMS text message 21 days after circumcision, (9) provided written informed consent, and (10) received a text confirming study enrollment.

### Recruitment

Urban recruitment occurred in the Gauteng province at 3 static VMMC sites in the Ekurhuleni district (Tembisa, Tsakane, and Katlehong North), each with its own VMMC team. Rural recruitment was conducted in the Bojanala district across outreach sites served by one VMMC team (Mogwase, Bafokeng, Bapong, and Letlhabile). All sites operated under routine VMMC service delivery provided by CHAPS and adhered to the NDoH VMMC guidance [36]. On the day of their VMMC, the participants were recruited; informed about the study on 2wT-based follow-up; and, if eligible and willing to participate, underwent informed consent by the study team at each site.

### Enrollment

All consented men were registered in a custom 2wT software app built using an open-source CHT. Consented men who did not receive text confirming study enrollment were immediately withdrawn from the study and resumed routine VMMC follow-up. Enrollment data included the name, phone number, and alternative phone number of the participants. Men were also asked to provide information on their wage (daily, weekly, monthly, or none) as well as the costs to come to the facility (food and transportation) for future consideration of patient VMMC costs.

### Randomization

A total of 1160 opaque security envelopes (580 intervention and 580 control) were used to randomize participants in a 1:1 ratio by following a randomized block design in groups of 10. Blocks were distributed equally between the urban and rural sites. This block design reduced variability within treatment conditions and potential confounding, allowing for interim analysis with near equal–size groups and more certain randomization within sites. The site coordinators were unaware of the envelope’s contents until the participant opened and revealed the group assignment to the coordinator. Randomization affected participant intervention only after routine VMMC surgical procedure and recovery. The study design required that the participants and clinic staff be aware of the allocation after the assignment.

### 2wT Training

The RCT study team, both researchers and implementation teams, was trained on 2wT through a 3-day hands-on 2wT study training session and an additional week of on-site support during the study launch at each site. For approximately 1 month, the 2wT system was reviewed daily by the central study team, providing brief feedback to the site teams via WhatsApp (Meta Platforms Inc) on areas for improvement. Throughout the RCT, the study team provided follow-up coaching and mentoring to the site teams via email, WhatsApp, and phone calls. The 2wT daily task sheets and quick guides provided reminders, whereas quarterly in-person refresher training ensured adherence to the 2wT study protocol. The study team provided additional support to the VMMC teams via weekly study calls, identifying problems and collaboratively resolving concerns with the 2wT system or VMMC program implementation. The study team visited the sites weekly for the first 3 months and monthly thereafter for quality improvement.

### Interventions

#### Overview

Most 2wT intervention activities in both routine and 2wT arms were maintained from the previous study [52]. Table 1 lists the major South African study activities by arm.

**Table 1:** Interventions for the 2-way texting (2wT) and routine arms.

Specific activities		Control	2wT
**Day 0 (day of VMMC^a^)**			
	Routine VMMC eligibility, consent, preoperative counseling, surgery, and completion of the NDoH^b^ client intake forms	✓	✓
	Study-specific consent	✓	✓
	Routine postoperative care, including wound care instructions, bandage removal guidance, and AE^c^ identification counseling	✓	✓
	Additional postoperative education on using the 2wT system, including review of labeled photos of potential AEs named in the daily SMS text message		✓
**Scheduled in-person follow-up**			
	Routinely scheduled day 2 and day 7 visits	✓	
	Study-specific day 14 visit (US $7 provided as phone credit)	✓	✓
	Active client follow-up for missed visits	Day 2 only	Day 14 only^d^
**2wT-specific processes**			
	Daily texts on days 1 to 13		✓
	Phone tracing if there is no SMS text message response by day 8		✓
**NDoH routine AE procedures**			
	In-person review for AE concerns on any day	✓	✓
	Emergency VMMC after-hours care	✓	✓
	AE identification, grading, management, and treatment	✓	✓
	AE reporting on routine NDoH forms	✓	✓

^a^VMMC: voluntary medical male circumcision.

^b^NDoH: National Department of Health.

^c^AE: adverse event.

^d^Day 14 active tracing for 2wT participants with whom there was no SMS text message, phone, or in-person contact from the day of VMMC.

#### Routine VMMC Care (Control Arm)

All VMMC care, from the assessment of AEs through complete healing, is provided free of charge by CHAPS in NDoH facilities. CHAPS follows all NDoH protocols based on the WHO guidelines and recommendations [29,36]. In the rural sites, according to standard operating procedures, 1 dedicated CHAPS VMMC team scheduled day 2 and day 7 postsurgery visits in response to either provider or patient considerations, either (1) meeting at a central, convenient location for the patient; (2) by sending a car to the patient’s home; or (3) by sending a driver to transport the patient to a central clinic location to meet the CHAPS VMMC team. In the urban settings, most patients independently returned for scheduled day 2 and day 7 visits, with only a few clients requiring transport support or home visits. In any setting, the patients could seek care outside scheduled visits at any Department of Health or private facility upon suspicion of AEs, but most patients returned to their VMMC site or contacted the CHAPS VMMC team for home visits. Sites used the standardized PEPFAR approach to identify, assess, and record the timing, type, and severity of AEs [48]. The COVID-19 pandemic disrupted VMMC service delivery several times over the course of the study, with travel restrictions and other infection prevention efforts resulting in a complete or partial reduction in scheduled follow-up visits, especially in rural areas. In both the urban and rural settings, the patients who missed the day 2 postsurgery visit were traced via phone call; if the patients missed both day 2 and day 7 visits and were not reachable by phone, up to 3 home visits were attempted to review the clients, when feasible.

#### 2wT Procedures (Intervention Arm)

**Figure 5:**
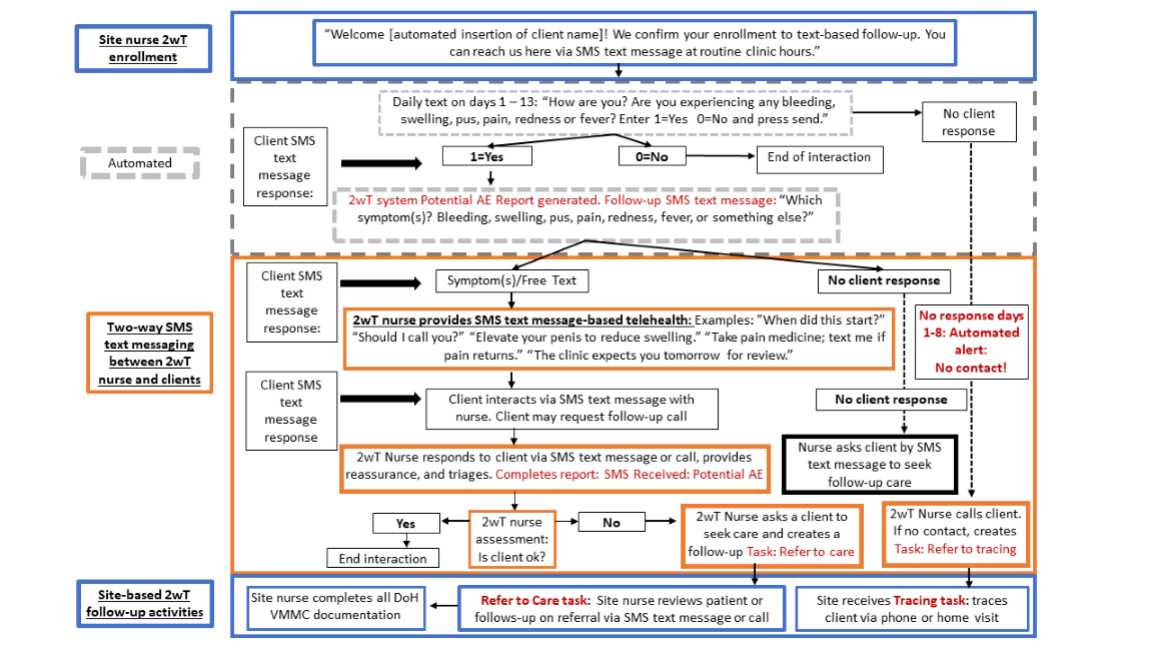
The 2-way texting (2wT) hybrid SMS text message flow (CC BY 4.0 [55]). AE: adverse event; VMMC: voluntary medical male circumcision.

On day 0 (day of VMMC), a 2wT study team member registered all the intervention participants in the app. The 2wT arm participants received up to 10 minutes of enhanced postoperative counseling using the 2wT flip-book on how to respond to the daily SMS text message and reviewed photos that illustrated the terms in the daily SMS text message: “How are you? Are you experiencing any bleeding, swelling, pus, pain, redness or wound opening? Enter 1=Yes, 0=No and press send.” The 2wT participants received the automated daily SMS text message on days 1 to 13 in 1 of 3 predominant languages of the area, English, Setswana, or isiZulu. The participants could respond via an SMS text message in any language. The clients were expected to respond to the daily SMS text message with a single numeric (0 or 1) response; they could respond to any daily SMS text message at any time. They could also initiate an unrestricted, freely-worded text response at any time, instead of or in addition to the daily numeric response. A *free text response* was any text-based SMS text message interaction with the VMMC nurse (eg, “Can I come to the clinic?” “I need some help” or “I’m in pain”). The 2wT nurse took no action for the participants who reported no concerns in their response to the daily SMS text message (0=no concerns). However, if a 2wT participant responded to any daily SMS text message with suspicion of a complication (1=yes concerns) or sent a free text reporting an issue, the 2wT VMMC nurse first interacted with the participant via SMS text message, providing education and reassurance. For participants with a potential AE or with need for further reassurance, the nurse triaged the participants, asking them to return to the clinic for a review (urban) or sending a clinical team to review the participants (rural) the following day or earlier if an emergency was suspected. The clients could request a call back to speak with the nurse at any time; the nurse could also initiate calls when desired. If the 2wT patients did not respond to texts on days 2 or 7, NDoH tracing was activated. The 2wT message flow is presented in Figure 5.

#### All Study Participants

The 2wT participants in both arms were asked to return to the clinic (urban) or had a visit arranged at their home or an outreach site (rural) for a study-specific, day 14 follow-up to review healing and verify AEs. Day 14 was chosen for verification because 95% of AEs are reported by day 14 [72]. On the day 14 visit, whether on time or delayed and whether the visit took place at the clinic, outreach site, or client’s home, a cell phone credit worth US $7 (100 Rands) was given to all the participants to compensate for time and travel. The control arm VMMC participants were not traced for missing the day 14 visit. The 2wT arm participants with whom there had been no contact by day 14 were actively traced via phone and home visits.

#### Power and Sample Size

The sample size was calculated based on the AE ascertainment (moderate or severe) occurring between the postoperative days 1 and 14. The noninferiority margin of −0.25% was set as the lower bound of a 95% CI for the maximum mean difference in the rate of cumulative AEs between the control and 2wT arms to conclude that 2wT is noninferior. This margin was set considering the mean AE rate of 0.5% in routine SSA VMMC programs at scale [40,42,46] and assuming that 2wT increases AE ascertainment to 2%, similar to the 1.9% AE ascertainment in the Zimbabwe RCT [50] and the widely accepted safety standard of 2% AE rate [35,48]. With a 10% loss to follow-up (LTFU), a sample size of 1104 men would provide at least 80% power to rule out a decrease in AE ascertainment of >0.25% based on the lower bound of the 1-sided 95% CI. The 1-tailed α value was set as.05.

#### Outcomes

The primary outcomes of interest were safety and workload. The primary safety outcome was the cumulative AE rate (moderate or severe) ≤day 14 and was calculated per arm as follows: (sum of moderate and severe AEs) / (total number of 2wT participants who had a clinic visit before or on day 14). Similar to the preceding RCT, the safety outcome of cumulative AE rate reported before or on the final study visit on day 14 included a range of 13 to 22 days after VMMC to provide a reasonable margin for participant delays [50]. The primary workload outcome was mean follow-up visits, which was calculated as the mean number of in-person visits, excluding the study-specific visit on day 14. The secondary outcomes included AE rates identified at the day 14 visit, SMS text message response (including daily response, 0=no AE or 1=potential AE, or any freely written text), AE timing, and the severity of AEs. The timing of actions resulting from potential AEs was described as a process measure for care continuity. Variables were created to capture in-person clinic visits and included the following ranges: *day 2 visit* included visits on days 1 to 4, *day 7 visit* included visits on days 5 to 10, and *day 14 visit* included days 13 to 22 after VMMC. All enrollment data on participant wage and visit costs were collected in South African Rand and converted to $US at the exchange rate of US $1=15.68 Rands (June 19, 2022).

#### Statistical Analyses

Data were exported from the 2wT web-based database and verified using the NDoH paper-record system. Descriptive statistics were calculated to describe the baseline characteristics and number of in-person clinic visits in each study arm. They included median and inter-quartile range (IQR) for the number of clinic visits and frequency for the number of in-person clinic visits. Cumulative AEs and the mean difference in proportions were compared by study arm using the Wilson score method and Manning score method, respectively, and 95% CI for proportions were calculated for both methods. The population for the primary safety outcome analysis (cumulative AEs) included all participants who made at least 1 visit between days 1 and 22. Secondary outcome analysis (AEs identified on day 14) included all participants who made a visit between days 13 and 22. The mean number of in-person clinic visits, excluding study visits, was compared between the 2 arms using a 2-tailed *t* test. The proportion of potential AEs was calculated as the number of potential AE responses divided by the total number of daily responses (no AE and potential AE). A superiority test was performed on the safety outcome using a 2-sided 95% CI and *P* value using Fisher exact test. The analyses were performed using Stata (version 17, StataCorp LLC) [73] and SAS (version 9.4, SAS Institute Inc) [74].

### Ethics Approval

This multiple principal investigator study was approved by the internal review boards of the University of Washington (study 00009703; principal investigator: CF) and the University of Witwatersrand, Human Research Ethics Committee (ethics reference number: 200204; principal investigator: GS). Written informed consent was obtained from all the participants before enrollment. A 5-person data and safety monitoring board was convened for this study and provided safety oversight, reporting at the conclusion of the study, on May 31, 2022, that the study was safely conducted according to the approved protocols.

## Results

### Enrollment and Demographics

The CONSORT (Consolidated Standards of Reporting Trials) flow diagram (Multimedia Appendix 1) shows the participant enrollment, assignment, follow-up, and analysis. Study recruitment began on June 7, 2021, and enrollment was completed on February 3, 2022. Follow-up concluded on February 21, 2022. In total, 1084 men were enrolled and randomized in the study. Among the 547 participants in the 2wT arm, 274 (50.1%) were from an urban setting, and 273 (49.9%) were from a rural setting. Of the 537 control participants, 269 (50.1%) were from an urban setting, and 268 (49.9%) were from a rural setting (Table 2). The median participant age was 31 (IQR 24-37) years. The majority of the participants (854/1084, 78.78%) opted for texts in the English language, followed by isiZulu (146/1084, 13.47%) and Setswana (83/1084, 7.66%). Vodacom (Vodacom Group Limited) was the most common mobile carrier (451/1084, 41.6%), followed by MTN (*MTN* Group Limited; 404/1084, 37.27%). Approximately 60.45% (648/1072) of the participants reported no wages. Among the 39.55% (424/1072) of participants who reported a wage, the median daily wage was US $19.1 (IQR US $12.76-US $28.70).

**Table 2:** Description of the voluntary medical male circumcision 2-way texting (2wT) cohort at enrollment by study arm.

Characteristic		Control (n=537)	2wT (n=547)	Full sample (N=1084)
Age (years), median (IQR^a^)		31 (25-36)	31 (24-37)	31 (24-37)
**Site, n (%)**				
	Urban	269 (50.1)	274 (50.1)	543 (50.1)
	Rural	268 (49.9)	273 (49.9)	541 (49.9)
**Language, n (%)**				
	English	429 (79.9)	425 (77.7)	854 (78.8)
	IsiZulu	71 (13.2)	75 (13.7)	146 (13.5)
	Setswana	37 (6.9)	47 (8.6)	84 (7.7)
**Mobile carrier, n (%)**				
	Cell C	55 (10.2)	49 (9)	104 (9.6)
	MTN	193 (35.9)	211 (38.6)	404 (37.3)
	Telcom	64 (11.9)	60 (11)	124 (11.4)
	Vodacom	224 (41.7)	227 (41.5)	451 (41.6)
	Other	1 (0.2)	0 (0)	1 (0.1)
**Daily wage (US $)^a^**				
	None, n (%)	333 (62.8)	315 (58.1)	648 (60.4)
	Yes, n (%)	197 (37.2)	227 (41.9)	424 (39.6)
	Value, median (IQR)	19.13 (12.76-29.34)	19.13 (12.76-28.70)	19.13 (12.76-28.70)
**Transport cost (US $)**				
	Value, median (IQR)	1.66 (0-2.55)	1.66 (0-2.30)	1.66 (0-2.55)
**Food cost (US $)**				
	None, n (%)	479 (89.2)	482 (88.1)	961 (88.7)
	Yes, n (%)	59 (11)	65 (11.9)	123 (11.3)
	Value, median (IQR)	1.50 (0.96-2.87)	1.28 (0.77-2.23)	1.28 (0.89-2.55)

^a^Information for wages was missing for 7 clients in the routine group and 5 clients in the 2wT group.

### 2wT Safety

Among participants who had an in-person visit by day 22 after VMMC, AEs were reported in 11 (n=477, 2.3%) 2wT participants and 5 (n=494, 1%) control participants (Table 3). The mean difference in proportions was +1.2% (95% CI −0.09 to +∞). As the lower limit of the 95% CI from the noninferiority comparison was above −0.25%, the 2wT intervention was noninferior to routine in-person follow-up care for AE ascertainment. The cumulative AE rates did not differ between the arms (*P*=.13). On the day 14 visit, 4 (0.9%) AEs were identified among the control participants and 1 AE (0.2%) was identified among the 2wT participants—a nonsignificant difference (*P=*.37).

**Table 3:** Comparison of combined moderate or severe adverse events (AEs) among circumcised males in South Africa by arm.

	Routine (n=537)			Two-way texting (n=547)			Difference in proportions, %		Noninferiority comparison		Superiority comparison	
	n	Proportion (95% CI)^a^, %	n		Proportion (95% CI)^a^, %			1-sided 95% CI^b^		2-sided 95% CI^b^		2-sided *P* value^c^
Cumulative AEs ≤day 14^d^	5/494	1.0 (0.4 to 2.3)	11/477		2.3 (1.3 to 4.1)	+1.2		−0.09 to +∞		−0.35 to 2.85		.13
AEs on day 14^e^	4/438	0.9 (0.4 to 2.3)	1/463		0.2 (0.03 to 1.2)	−0.7		N/A^f^		−1.61 to +∞		.37

^a^CI for binomial proportions were calculated using the Wilson score method.

^b^CI in binomial proportions were calculated using the Farrington–Manning score method.

^c^*P* value comparison group is by Fisher exact test.

^d^Cumulative moderate and severe AEs reported during study visits from days 1 to 22 among the participants with at least 1 postoperative visit.

^e^Cumulative moderate and severe AEs reported during study visits from days 13 to 22 among participants.

^f^N/A: not applicable.

### Follow-up Visit Workload

The texting intervention group reported a mean of 0.22 (SD 0.65) nonstudy visits per person (median 0) compared with 1.34 (SD 0.85) nonstudy visits per person (median 2) in the control group over the 14-day postoperative period—a significant difference of 1.12 visits between the means (*P*<.001; Table 4). There was an 84% reduction in the follow-up visit workload using 2wT compared with routinely scheduled visits. The participants in the control group were each expected to return for 2 scheduled visits; they returned for 719 visits, total (median 2, IQR 1-2), excluding the study-specific day 14 visit. Among the 537 control participants, 105 (19.6%), 159 (29.6%), and 266 (49.5%) returned for 0, 1, and 2 visits, respectively, and 432 (80.4%) returned for at least 1 visit, excluding the study-specific visit. Among the 2wT participants, there were 118 visits, total (median 0, IQR 0-0), not including the study-specific day 14 visit. Among the 547 participants in the 2wT arm, 60 (11%) and 18 (3.3%) returned for 1 and 2 visits, respectively (Table 3). Excluding the day 14 visit, most 2wT participants (n=464, 84.8%) did not return for any postoperative visits.

Comparison within arms (stratified by location [urban vs rural], not shown) identified similar, significant differences in workload between arms. Among the control participants only, visit attendance was nearly 10% higher among the urban participants than among the rural participants on day 2 (200/269, 74.3% vs 175/268, 65.3%; *P*=.02). LTFU was also significantly higher among the urban participants (23/269, 8.6%) than among the rural participants (6/268, 2.2%) in the control arm (*P*=.002). Among the 2wT participants only, more urban participants (28/274, 10.2%) than rural participants (11/273, 4%) made a visit on day 2 (*P*=.01). LTFU was higher among the urban 2wT participants but not significantly (30/274, 10.9% vs 17/273, 6.2%; *P*=.07). The follow-up visit workload reduction was 79% in the urban setting and 89% in the rural setting.

**Table 4:** Comparison of the workload outcome by arm (excluding the study-specific day 14 visit).

		Control (n=537)	2wT^a^ (n=547)	Difference in means (95% CI)		2-sided *P* value	
**Number of clinic visits^b^**							
	Value, mean (SD)	1.34 (0.85)	0.22 (0.65)	1.12 (1.03-1.21)		<.001	
	Value, median (IQR)	2 (1-2)	0 (0-0)	N/A^c^		N/A	
Males with ≥1 in-person follow-up visits within 14 days^d^, n (%)		431 (80.3)	83 (15.2)	N/A		N/A	
**Total visits per client, n (%)**					N/A		N/A
	0	105 (19.6)	464 (84.8)				
	1	159 (29.6)	60 (11)				
	2	266 (49.5)	18 (3.3)				
	3	4 (0.7)	3 (0.5)				
	4	1 (0.2)	1 (0.2)				
	5	1 (0.2)	0 (0)				
	6	0 (0)	0 (0)				
	7	1 (0.2)	0 (0)				
	8	0 (0)	0 (0)				
	9	0 (0)	1 (0.2)				

^a^2wT: 2-way texting.

^b^There were a total of 719 clinic visits in the control group and 118 clinical visits in the 2-way texting group.

^c^N/A: not applicable.

^d^The US President’s Emergency Plan for AIDS Relief indicator.

### Secondary Process Outcomes

#### Texting Response Rates

**Figure 6:**
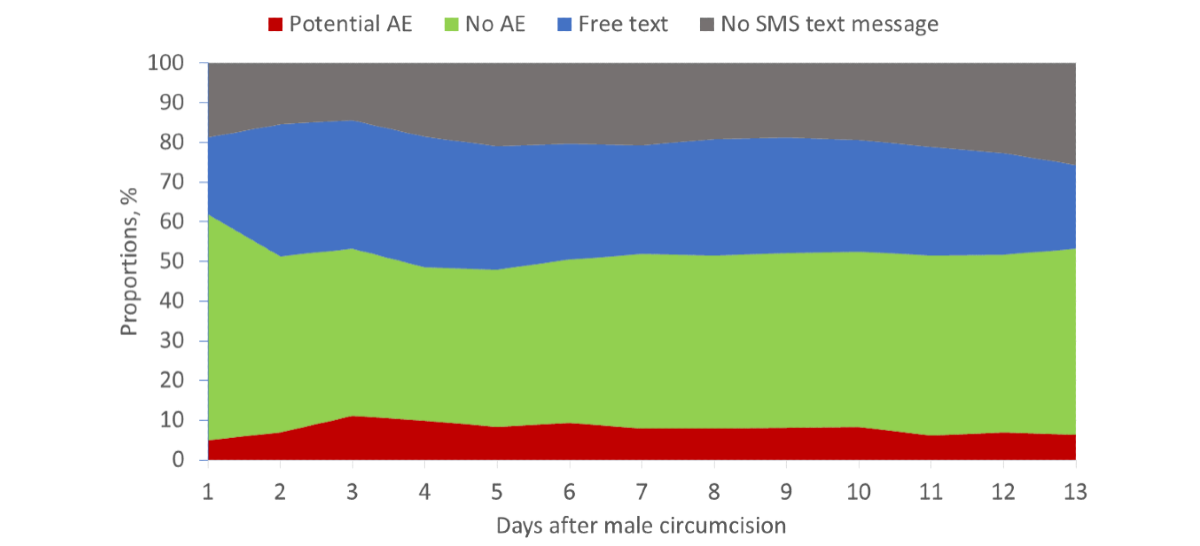
Daily SMS text message responses among 2-way texting participants. AE: adverse event.

Response rates were high among the 2wT participants, ranging from a maximum of 86% (468/547) on day 3 to a minimum of 74% (407/547) on day 13. On days 2 and 7, 85% (463/547) and 79% (433/547) of the participants responded, respectively. Among the 547 participants, 514 (94%) responded at least once in 13 days. Only 6.4% (33/514) participants never responded to a daily text. Of the 547 participants in the 2wT arm, 301 (55%) sent at least 1 potential AE text over the 13 days. On an average day, 44% (241/547) responded with “No AE,” 8% (44/547) responded with “potential AE,” 28% (153/547) responded with a free text, and 20% (109/547) did not respond (Figure 6). Among those with an expected 0 or 1 response to the daily SMS text message (no AE or potential AE), a mean of 15% (n=43) responded with a potential AE each day. On day 3, a total of 21% (115/547) of the participants responded with a potential AE, but only 12% (66/547) responded similarly on day 13.

#### Timing and Severity of AEs

Among the 2wT participants, there were a total of 11 AEs, with 9 moderate (2 bleeding, 4 infections, and 3 wound disruptions) and 2 severe bleeding AEs (Table 5). Among the control participants, there were a total of 5 AEs, all moderate (2 infections, 2 wound disruptions, and 1 other [swelling]). The 2wT participants were identified with AEs earlier than the control participants. Among the 2wT participants, 64% (7/11) AEs were identified on or before day 7, whereas only 20% (1/5) AEs among the control participants were identified during this early period. Late AEs (>day 14) were identified among 2 (0.4%) out of 537 control participants. All AEs were related to VMMC, and all occurred on or before day 22. Mild AEs were not included in the study outcomes.

**Table 5:** Adverse event (AE) severity and type by arm, postoperative day, and location.

AE severity	Postoperative day	AE type	Study arm			Location	
			2wT^a^ (n=11)	Control (n=5)	Urban (n=14)		Rural (n=2)
Severe	1	Bleeding	✓				✓
Severe	3	Bleeding	✓		✓		
Moderate	2	Bleeding	✓		✓		
Moderate	2	Bleeding	✓		✓		
Moderate	4	Infection	✓		✓		
Moderate	5	Infection	✓		✓		
Moderate	7	Infection	✓		✓		
Moderate	7	Swelling		✓	✓		
Moderate	10	Wound disruption	✓		✓		
Moderate	10	Wound disruption	✓				✓
Moderate	12	Infection	✓		✓		
Moderate	14	Infection		✓	✓		
Moderate	14	Wound disruption		✓	✓		
Moderate	14	Wound disruption	✓		✓		
Moderate	17	Wound disruption		✓	✓		
Moderate	22	Infection		✓	✓		

^a^2wT: 2-way texting.

#### Time Between Potential AE Text and Follow-up

Among the 2wT participants, there were 7111 responses to the daily SMS text message, of which 559 (7.9%) responses were a 1, or a potential AE. The nurse completed 704 potential AE forms, which included reports stemming from men who conveyed issues or concerns first via a free text and not the daily response; 649 potential AE forms were completed and were unique to 1 client per day. Potential AE forms included the documentation of concerns (AE type or severity) and triage (education, reassurance, wound care instructions, etc). The nurse could refer with or without suspicion of an AE, allowing them flexibility to assess each client individually. Referrals to care created a task for the site team to complete an in-person review. From the 649 filled potential AE forms, 50 (7.7%) referrals were made (including for 3 moderate and 1 severe potential AEs): 32 returned within 3 days, and 4 AEs were ascertained (moderate: 3/4, 75% and severe: 1/4, 25%).

#### LTFU Rate

There were 47 (8.6%) out of the 547 participants in the 2wT arm who did not return for any in-person follow-up and were considered LTFU; of the 47 participants, 45 (96%) were successfully contacted by phone and reported no concerns, 1 (2%) had a physical visit before day 13 and was observed to be healing on schedule, and 1 (2%) had no postoperative contact. Among the 537 control participants, 29 (5.4%) were LTFU; of the 29 participants, 10 (34%) were successfully contacted by phone and reported no concerns, 2 (7%) had visits, and 17 (59%) had no postoperative contact.

## Discussion

### Principal Safety and Workload Findings

The study conclusively demonstrated that in South Africa, 2wT for VMMC follow-up is noninferior to in-person reviews for AE ascertainment, providing rigorous evidence in support of 2wT safety in routine settings. The 2wT approach also dramatically reduced in-person visits, allowing the clinical team to focus on, and care for, those with potential complications rather than conducting scheduled follow-ups for all men, demonstrating clear efficiency gains. Most 2wT participants interacted via 2wT and elected to heal independently at home rather than return for an in-person review. The South African 2wT RCT adhered to best practices in the monitoring and evaluation of digital health innovations [14,70,75], adding to the evidence from both the Zimbabwe RCT and routine scale-up that 2wT is as safe as, and more efficient than, routine in-person reviews for postoperative follow-up [50,55]. The 2wT results also indicate some of the advantages of telehealth for safe and efficient surgical follow-up [76-78]. As this pragmatic RCT took place within NDoH VMMC settings and largely with routine HCWs, these results strongly suggest that the 2wT telehealth approach to VMMC follow-up should be rolled out as an option for providers and patients. We discuss several advantages of and barriers for 2wT to scale successfully.

First, recent findings from usability testing among 2wT HCWs [79] showed that 2wT responded well to the expressed needs of HCWs and clients, increasing the likelihood of sustained use and widespread adoption [80,81]. From inception, 2wT adaptation, testing, and optimization processes adhered to the common principles for mHealth optimization focused on both patient and provider users, the engagement of broad stakeholders, providing training (and refresher training) to users via hands-on methods, using a simple technology with a familiar user interface, addressing a recognized need for updated VMMC policy, and using widely available SMS text messaging technology [82]. The 2wT approach complements the efficiency of automation with alerts for nurses to engage in direct client messaging when needed, thereby enhancing the nurse-patient relationship. Nurses who refer clients to care trigger an alert for follow-up, requiring an HCW to document subsequent visits, confirm patient-reported improvement, or initiate additional tracing efforts. The embedded system alerts escalate clients without responses to tracing and remind providers to close referral loops when clients are found. The engagement of an SMS text message aggregator in South Africa also increased the efficiency of automated SMS text messages, easing the identification and resolution of any systematic message delays or failures and enhancing user experience. With 2wT’s inclusion of core CHT features, such as longitudinal patient records, task management, and reporting support, 2wT adaptation could transform other postoperative or other acute follow-ups outside the VMMC context.

The 2wT approach improved the quality of postoperative follow-up, as evidenced by consistent and correct AE identification, a standard indicator of quality VMMC postoperative care. Improvements are likely owing to both patient and provider engagement. In 2wT, 5 moderate AEs were identified on or before day 7, after the men were first triaged by the 2wT nurse. This early AE ascertainment suggests that direct 2wT communication between the nurse and the patient encouraged men to return promptly for review when needed. By contrast, 4 men in the control group arm were identified with AEs at the day 14 visit or later, suggesting that they may have delayed care, or not received care, in the absence of the study. This care-seeking behavior suggests that 2wT improves patient health literacy and care engagement, placing the responsibility for healthy healing into the patient's hands and shifting the power balance toward the individual rather than the formal health sector. Furthermore, within the study context, providers promptly documented AEs, including the cascade from potential AEs, through AE clinical management. Although AE ascertainment is a priority indicator of quality VMMC care [35], AEs are chronically underreported in routine settings [45,46,51], including in South Africa [40,44]. The 2wT approach appears to improve AE ascertainment and reporting, contributing to improved patient management and program outcomes.

Similar to the findings from Zimbabwe that showed that 2wT improved efficiency and reduced costs [53], 2wT reduced the follow-up visit workload in South Africa by 79% in urban and 89% in rural settings, leading to program cost savings in both settings [83]. These visit reductions were observed in the already-reduced in-person visits implemented for COVID-19 precautions. Whether VMMC follow-up visits return to prepandemic levels, reducing unnecessary visits would likely have a greater impact in rural settings where the VMMC service model is predominantly outreach, reducing travel time. The benefits of fewer visits could also have a positive impact on VMMC client demand, reducing barriers to VMMC such as transportation costs or time away from work [84]. Moreover, in the future, 2wT could strengthen the continuum of referrals even outside participating VMMC clinics or clinicians by engaging directly with patients. Participants enrolled in 2wT in a central location could seek care at NDoH sites outside the 2wT catchment area and self-report visits, AE diagnoses, and treatments via 2wT. This information pathway could further contribute to improved AE reporting and visit documentation while reducing the burden on HCWs. Advocating for formal PEPFAR approval of 2wT-based follow-up in lieu of in-person visits could fulfill the PEPFAR indicator of at least 1 postoperative visit within 14 days [3] while simultaneously reducing the HCW burden and improving patient care.

Despite these benefits, there are challenges to scaling 2wT successfully. First, digital literacy among HCWs remains low [85]. Patients only need to be comfortable with basic SMS text messaging technology, but HCWs interacting with 2wT must be able to manage the app at a comparable level to WhatsApp, a nonnegligible challenge in some settings or for some HCWs. Patient demand must also increase. For success, 2wT education and outreach are needed to raise awareness around 2wT advantages for both quality care and reduced patient costs [86]. In addition, some patients may lack confidence in telehealth owing to persistent network and infrastructure challenges [87], stymying 2wT growth. The 2wT approach also suffers, in part, by a perception of increased workload. HCWs save time by conducting far fewer routinely scheduled follow-up reviews and by reducing travel for the reviews. However, that time gained is reallocated to interactions with men via 2wT and to other routine duties. The time and cost savings of 2wT are likely to be more appreciated at the health services level as overall efficiency gains across VMMC or routine service delivery, and not as reduced workload during duty hours. As with all postoperative care, patients may require care at any time. Although clients were informed that 2wT is not for emergencies, nurses were required to review 2wT SMS text messages in evenings and on weekends, an issue that should be addressed via the rotation of duties and flexible work hours. Moreover, the 2wT dashboards and data analytics support routine VMMC program reporting. However, approvals of digital documentation for the NDoH and implementing partners, in lieu of and not in addition to paper reporting, will be needed to facilitate scale-up. Finally, as with all digital health interventions, there are concerns regarding sustainability. However, as 2wT specifically targets patients in need of a brief but intensive interaction with a provider, and not a repeated interaction with a health care unit, the benefits of immediate scale-up offset the concerns for long-term eHealth integration.

### Limitations

This study has several limitations. As for clients, men without their own phones or those who shared phones were excluded. The 2wT approach would also not work in remote settings without at least some cell connectivity. Using SMS text messaging for 2wT makes it more equitable and accessible for those with basic phones [20] but could reduce uptake for those with feature or smartphones or those who prefer WhatsApp. The 2wT automated SMS text messages were only scripted in 3 predominant languages of the area, potentially excluding men whose preferred language was not offered. Notifiable AEs could be underreported in either group. Both arms received enhanced AE identification education; however, men in the 2wT arm received additional counseling using illustrative photos to explain the words of the daily SMS text message, a bias that could have increased AE identification in the 2wT arm. The RCT study team provided quality assurance throughout the study to ensure adherence to the protocols, increasing supervision and support. However, continued success with 2wT in Zimbabwe within routine, nonresearch settings suggests that 2wT would have similar results outside RCT structures. Complementary usability assessment from both HCW and client perspectives and costing results are forthcoming and, therefore, not included in this paper. Finally, in the context of the COVID-19 pandemic, this RCT emphasized pragmatism and flexibility, a limitation that also facilitated the implementation of the study in challenging times. During the RCT enrollment period, there were 3 waves of the COVID-19 pandemic, causing various levels of clinic closures, service delivery restrictions that affected follow-up, and increased turnover in health care personnel. During the COVID-19 pandemic, the PEPFAR guidelines were changed to allow for phone-based VMMC follow-up when feasible [88]. As a result, in-person visits in the control group likely declined by an unknown percentage. Even in this context, the 2wT study identified a significant reduction in follow-up visit workload with maintained quality of care, reinforcing these dual advantages of 2wT for routine VMMC settings.

### Conclusions

Over 4 years of rigorous implementation science research and evaluation in both South Africa and Zimbabwe, 2wT-based telehealth successfully provided quality VMMC follow-up for over 24,000 males, aged ≥15years, in both rural and urban settings [50,53-55]. These studies respond to global calls for robust evidence generation in digital health interventions [82,89,90] and support the recent global VMMC policy changes allowing for the use of postoperative telehealth, in lieu of scheduled visits, for low-risk post-VMMC clients [88]. The 2wT care model, specifically, improved more than safety. The 2wT approach motivated males to actively assume care-seeking responsibility for potential AEs, strengthening relationships between males and nurses and creating a partnership in healing. The 2wT approach also added value in the context of the COVID-19 pandemic by providing dual SMS text messaging–based telehealth and infection prevention benefits. SSA is ready for a 2wT telemedicine approach based on widespread SMS text messaging technology. Therefore, in recognition of scarce health care resources and strong evidence from this rigorously tested intervention, 2wT should be scaled in both rural and urban VMMC programs. Further consideration of simple telehealth approaches such as 2wT could also provide powerful pathways to improve care and reduce workload beyond VMMC, bringing similar benefits for other acute follow-up care contexts, including COVID-19, acute respiratory infection, and other short-term postoperative care.

## Appendix

Multimedia Appendix 1 CONSORT eHealth Checklist.

## Abbreviations

2wT: 2-way texting
AE: adverse event
CHAPS: Centre for HIV-AIDS Prevention Studies
CHT: Community Health Toolkit
CONSORT: Consolidated Standards of Reporting Trials
HCW: health care worker
LTFU: loss to follow-up
mHealth: mobile health
NDoH: National Department of Health
PEPFAR: US President’s Emergency Plan for AIDS Relief
RCT: randomized controlled trial
SSA: sub-Saharan Africa
VMMC: voluntary medical male circumcision
WHO: World Health Organization

## Data Availability

The data sets generated in the randomized controlled trial and analyzed during this study, including demographics, SMS text messaging response rates, clinic visits, and adverse events, are available from the corresponding author upon reasonable request.
